# Association of lumbar spine stiffness and flexion-relaxation phenomenon with patient-reported outcomes in adults with chronic low back pain – a single-arm clinical trial investigating the effects of thrust spinal manipulation

**DOI:** 10.1186/s12906-017-1821-1

**Published:** 2017-06-09

**Authors:** Ting Xia, Cynthia R. Long, Robert D. Vining, Maruti R. Gudavalli, James W. DeVocht, Gregory N. Kawchuk, David G. Wilder, Christine M. Goertz

**Affiliations:** 1 Palmer Center for Chiropractic Research, Palmer College of Chiropractic, 741 Brady Street, Davenport, IA 52803 USA; 2 Department of Physical Therapy, University of Alberta, Edmonton, AB Canada; 3 Department of Biomedical Engineering, University of Iowa, Iowa City, Iowa, USA

**Keywords:** Chronic low back pain, Complementary and alternative medicine, Spinal manipulation, Disability, Pain intensity, Spinal stiffness, Flexion-relaxation phenomenon, Instrument-assisted assessment

## Abstract

**Background:**

Spinal manipulation (SM) is used commonly for treating low back pain (LBP). Spinal stiffness is routinely assessed by clinicians performing SM. Flexion-relaxation ratio (FRR) was shown to distinguish between LBP and healthy populations. The primary objective of this study was to examine the association of these two physiological variables with patient-reported pain intensity and disability in adults with chronic LBP (>12 weeks) receiving SM.

**Methods:**

A single-arm trial provided 12 sessions of side-lying thrust SM in the lumbosacral region over 6 weeks. Inclusion criteria included 21–65 years old, Roland-Morris Disability Questionnaire (RMDQ) score ≥ 6 and numerical pain rating score ≥ 2. Spinal stiffness and FRR were assessed pre-treatment at baseline, after 2 weeks and after 6 weeks of treatment. Lumbar spine global stiffness (GS) were calculated from the force-displacement curves obtained using i) hand palpation, ii) a hand-held device, and iii) an automated indenter device. Lumbar FRR was assessed during trunk flexion-extension using surface electromyography. The primary outcomes were RMDQ and pain intensity measured by visual analog scale (VAS). Mixed-effects regression models were used to analyze the data.

**Results:**

The mean age of the 82 participants was 45 years; 48% were female; and 84% reported LBP >1 year. The mean (standard deviation) baseline pain intensity and RMDQ were 46.1 (18.1) and 9.5 (4.3), respectively. The mean reduction (95% confidence interval) after 6 weeks in pain intensity and RMDQ were 20.1 mm (14.1 to 26.1) and 4.8 (3.7 to 5.8). There was a small change over time in the palpatory GS but not in the hand-held or automated GS, nor in FRR. The addition of each physiologic variable did not affect the model-estimated changes in VAS or RMDQ over time. There was no association seen between physiological variables and LBP intensity. Higher levels of hand-held GS at L3 and automated GS were significantly associated with higher levels of RMDQ (*p* = 0.02 and 0.03, respectively) and lower levels of flexion and extension FRR were significantly associated with higher levels of RMDQ (*p* = 0.02 and 0.008, respectively) across the 3 assessment time points.

**Conclusions:**

Improvement in pain and disability observed in study participants with chronic LBP was not associated with the measured GS or FRR.

**Trial registration:**

NCT01670292 on clinicaltrials.gov, August 2, 2012

## Background

Low back pain (LBP) has long been recognized as a major health problem due to its high prevalence rate, negative impact on quality of life, and staggering socioeconomic costs [1–3]. Spinal manipulation (SM) is a commonly used form of manual therapy and has been recommended by recently published clinical practice guidelines for LBP management [4–6]. Despite the demonstrated benefits [7, 8], the mechanisms linking SM to LBP improvement are largely unknown.

Manual assessment of the spine is routinely employed by manual therapists to evaluate patients and their response to treatment [9–11]. Typically, clinicians describe their findings in terms of perceived segmental motion [12, 13]. Different from the perceived motion, stiffness in this study is defined as the quantitative measure of the resistance to motion. It is postulated that restoring normal spinal stiffness and mobility facilitates spinal function improvement and pain reduction [14–16]. One form of spinal stiffness assessment is accomplished by applying anteriorly oriented pressure over a spinal segment of a prone lying patient while manually perceiving resistance or mobility [17]. Instrument-assisted methods [14, 18–20], as well as stiffness reference-based protocols [21–23], have been developed to assess spinal stiffness. In this study we used 3 instrument-assisted methods: i) hand palpation, ii) a hand-held instrumented device, and iii) an automated indenter device, to obtain force-displacement curves and subsequently lumbar spine stiffness. The hand palpation method was chosen because of its reported use in clinical practice [12, 13]. The hand-held stiffness measurement device was developed in our laboratory and demonstrated ease of use and good reliability on patients with LBP [18]. The automated indenter was used for its precision control and potentially more accurate measurement and has demonstrated clearly its value in assessing spinal stiffness in patients with acute LBP [20, 24, 25].

The association between LBP and spinal stiffness is complex [26, 27]. Shirley and Lee (1993) applied oscillating posterior-anterior (PA) pressures to L3 and L4 and found patients with LBP had a higher spinal stiffness than those were asymptomatic [28]. Brodeur and Delre (1999) applied a similar technique and found that male patients with LBP had higher spinal stiffness than asymptomatic males [29]. Owens et al. (2007) applied PA pressure at individual lumbar segment levels using a hand-held device but found little agreement between the severity or chronicity of the LBP complaint and spinal stiffness, nor between the stiff or tender segments assessed by clinicians and the stiffness measurements from the probe [30]. Further, spinal stiffness was observed to decrease after treatment, though the association between change in spinal stiffness and change in clinical outcomes was either not examined [14, 21, 24], statistically significant [20], or not statistically significant [22].

Spinal stiffness is in part maintained through active contraction of the spine musculature. Pain is known to interfere with both afferent and efferent aspects of neuromuscular control [31–34], hence may affect spinal stiffness. One example of normal neuromuscular control is the flexion-relaxation phenomenon (FRP), during which the lumbar paraspinal muscles relax when the trunk assumes a fully flexed position. However, individuals with LBP often have elevated lumbar muscle activities in the fully flexed position and consequently do not demonstrate the FRP, or at least not as distinctly [35, 36]. Examining FRP in individuals with LBP undergoing treatment may provide useful information regarding the functional status of trunk musculature and the contribution to spinal stiffness. To our knowledge, this study is the first to attempt to identify an association between FRP measures and patient-report outcomes in patients with chronic LBP receiving SM. Neblett et al. (2003) proposed that the baseline flexion-relaxation ratios (FRRs) combined with baseline lumbar range of motion values could be associated with which type of treatment results in the most beneficial clinical outcomes for specific individuals with LBP [37]. Although not tested for clinical validity, it was theorized that if a LBP sufferer displays normal FRP as well as normal lumbar range of motion, that individual may benefit from receiving earlier strength exercises thereby avoiding the need for a time-consuming mobilization period and lowering treatment duration and cost. Mayer et al. (2009) reported that a majority of patients with chronic disabling occupational lumbar disorders failed to demonstrate a normal FRP but did so after completing an interdisciplinary functional restoration program [36]. Kim et al. (2013) recently observed an increase in FRR in both arms of a trial on patients with chronic LBP [38]. Specifically one group received usual physical therapy and the other group performed a lumbar stabilization exercise using the Neurac sling after receiving ordinary physical therapy. Another study showed that the FRR of patients with chronic LBP increased immediately after a single treatment of spinal manipulation [39].

The primary objective of the current study was to examine the association of lumbar spine stiffness and FRP (i.e., physiological variables) with patient-reported pain intensity and disability in adults with chronic LBP who received twice-weekly side-lying, high-velocity, low-amplitude thrust SM over 6 weeks. The findings would help us to understand potential acting mechanisms of SM on patients with chronic LBP (e.g., through the effect on spinal stiffness and/or FRR), as well as clinical values of lumbar spine stiffness and FRP assessments in chronic LBP management.

## Methods

### Study population

Participants were recruited from local communities (Davenport, IA, Bettendorf, IA, Rock Island, IL, Moline, IL and the surrounding areas) primarily through direct mail advertisements. Eligible participants were 21–65 years old, met the Quebec Task Force for Spinal Disorders (QTF) classification of 1, 2, or 3 [40], reported LBP duration for more than 12 weeks, scored ≥6 on the Roland Morris Disability Questionnaire (RMDQ) during a voice phone screen conducted by study personnel before the first baseline visit, a patient-reported average pain over the past 24 h ≥ 2 on an 11 point numerical rating scale at phone screen and baseline visits 1 and 2 (BL1 and BL2), and an average pain ≥4 either at phone screen or BL1. More detailed descriptions of the inclusion/exclusion criteria and the screening process can be found in the study protocol [41]. Ethics approval was obtained from the Palmer College of Chiropractic Institution Review Board (IRB) with the Assurance Number - X2011X141. Consent was obtained from participants using the approved informed consent documents during BL1.

### Study design

The current study was designed as a 6-week, single-arm clinical trial using a side-lying, thrust SM to treat participants with chronic LBP at a twice weekly frequency. Demographic and health status measures were collected during BL1. The physiological measures analyzed included posterior-anterior stiffness of the lumbar spine and lumbar spine FRR collected immediately prior to SM treatment at treatment visit 1 (TV1), TV5, and TV12. The primary patient-reported outcomes included pain intensity and disability, collected at BL1 and prior to SM treatment at TV6 and TV13. For simplicity, baseline, after 2 weeks, and after 6 weeks outcomes denoted these data collection time points as there were roughly 2 TVs per week. All study activities were carried out in the Research Clinic, Palmer Center for Chiropractic Research in Davenport IA, United States. This study is reported according to the Consolidated Standards of Reporting Trials guidelines [42].

### Spinal manipulation intervention

We chose a commonly used side-lying, high velocity low amplitude thrust SM referred to as Diversified chiropractic technique to treat LBP [43, 44]. In brief, participants assumed a lateral and relaxed recumbent position with the lower extremity straightened and the upper knee and hip flexed and slightly adducted across midline. The study clinician, standing and facing the participant, stabilized the participant’s shoulder or upper arm with one hand while the participant’s flexed leg was stabilized with the clinician’s thigh. With heel or pisiform area of the opposite hand, the clinician delivered a quick and controlled low-amplitude thrust. The manipulation target areas were restricted to the lumbar spine and sacroiliac joints. Potential spinal segmental contact points determined by the clinician at each visit included mammillary process, spinous process, and intervertebral space. Pelvic segmental contact points included posterior-superior iliac spine, ischial tuberosity, sacral base, sacral apex, sacral ala and lateral to the sacral ala. Study clinicians determined the target vertebral or pelvic segments and contact points based on varied clinical information such as the primary working diagnosis, positions of provocation and relief, tenderness, prior response to treatment, and tolerance. Because symptom characteristics and clinical findings sometimes changed throughout the course of care, segments targeted for manipulation were not always identical between visits. Stiffness measures used in this study did not inform clinical decision-making. No SM procedures involved a pulling motion and participants were not asked to resist the SM position. The number of manipulations and specific target joints were determined by the clinician at each individual visit. It is noteworthy that during TV1, TV5, and TV12, modified side-lying thrust SM was used as the intervention to facilitate segmental load estimation (to be reported elsewhere). SM procedures applied during these visits involved stabilizing the participant’s shoulder with a strap instead of the clinician’s hand. The clinician’s stabilizing hand was placed on a handle located just above, but not contacting, the participant’s shoulder. Details of the modified thrust SM can be found in the study protocol [41].

SM was applied by 3 study clinicians, each with at least 15 years of experience in using this intervention. Clinicians had experience in general clinical practice, teaching SM procedures, and in clinical trial research settings. Clinicians and biomechanics personnel training occurred weekly for several months before study implementation to ensure appropriate treatment could be administered in a manner that also allowed data collection during TV1, TV5, and TV12.

### Patient-reported outcomes

The primary patient-reported outcomes included the Visual Analog Scale (VAS, 100 mm horizontal scale, 0 = no pain; 100 = worst imaginable pain) for LBP intensity and the 24-item RMDQ for LBP-related disability. The specific anchor for VAS was the average LBP over the past 24 h. Both instruments are commonly used in LBP studies and have been well validated [45–49].

### Physiological measures

#### Posterior-anterior spinal stiffness over the spinous processes of the lumbar spine

Lumbar spine stiffness was assessed with participants in a prone position using three methods sequentially: i) hand palpation, ii) a hand-held instrumented device, and iii) an automated indenter device [41]. In brief, during hand palpation, the participants laid face down on an instrumented treatment table when the clinician applied anteriorly directed force over each lumbar segment sequentially from L5 to L1. Force was recorded using force plates (Model 4060-NC, Bertec Inc., Columbus, OH) embedded in the treatment table. Movement of the hand was monitored using an infrared marker (Optotrak 3020/Certus hybrid system, Northern Digital Inc., Waterloo, Ontario, Canada) placed at the doctor’s wrist. Participants breathed normally during the test. Though palpation can be applied over other areas, such as the zygapophyseal joints, spinous process contact best approximated other measures used in this study and are consistent with how some clinicians manually assess spinal stiffness [12, 13].

For the hand-held device method [18], force was measured using a force transducer (Model # LC201–50, Omegadyne Inc., Sunbury, OH) while movement was monitored using infrared markers. Participants were lying prone during the test and instructed to first inhale deeply, exhale fully, again inhale half way and then hold their breath before the assessment initiated and during the test. The hand-held device was manually pressed on a lumbar spinous process anteriorly to a maximum force of 80 N for 5 cycles at a rate of approximately 1 repetition per second. The average stiffness obtained over the last 4 cycles was used for analysis [18, 50]. The described procedure demonstrated good reliability at 0.79 (95% confidence interval, 0.739–0.832) in patients with subacute or chronic LBP [18]. The 80 N limit was chosen because higher loads are more likely to produce discomfort but not improve stiffness calculations. Each of 5 lumbar segments was tested in the same manner, though the order of the sequence (e.g., L5, L2, L3, L1, L4) was randomly generated by computer immediately prior to measurement at each visit (TV1, TV5, and TV12) for each participant. Because this test created a subtle mobilization of the lumbar spine due to repeated pressure, random sequencing was used to avoid the potential for the testing order to influence stiffness measurements.

For the automated device method [19, 25], a programmable indenter applied force anteriorly up to 60 N with a force feedback control. The advancement rate of the indenter was 2.5 mm per second. The indenter was positioned over the spinous process nearest the most anterior point of the lumbar curve (typically at L3) of a participant lying prone. Participants were instructed to hold their breath at the end of exhalation before the assessment initiated and during the test. After a testing round, the procedure was repeated 3 times with the average of three trials used for analysis. The described procedure demonstrated good reliability at 0.98 (95% confidence interval, 0.93–0.99) in asymptomatic individuals and 0.98 (95% confidence interval, 0.94–0.99) in participants with LBP [19].

Global stiffness (GS) was calculated as the slope of the force-displacement curves over the force range from 10 N to 60 N for all 3 spinal stiffness assessment methods. GS variation (GSV) over the entire lumbar spine and normalized GSV (nGSV) were further computed from GS obtained at L1 to L5 during the hand palpation and the hand-held device methods. Details of GS, GSV, and nGSV can be found in the study protocol [41]. GS obtained using the automated indenter method at the most anterior point of the lumbar curvature, GS obtained during the hand palpation at L3, and GS obtained using the hand-held device method at L3 were used.

#### Flexion-relaxation phenomenon

Surface electromyography (EMG) measurements of lumbar paraspinal muscle activity for FRP analysis were taken using a Therapeutics Unlimited system (model 544, Iowa City, IA) with an electrogiometer for motion detection for the first 30 participants and then a Trigno wireless system with imbedded trial-axial accelerometer (Delsys Inc., Natick, MA) for the remaining participants. The change in EMG measurement systems was due to failure of the electrogoniometer in the original setup. During EMG assessment, participants were instructed to bend forward as far as possible while keeping the knees straight, holding the flexed position for 3 s, then returning to the upright standing position. This movement pattern was repeated 3 times at a pace preferred by participants. To quantify the degree of FRP, 4 different measures of FRR including flexion FRR, extension FRR, asymmetry in flexion FRR, and asymmetry in extension FRR were extracted from EMG signals as described in the study protocol [41].

### Sample size

A minimum sample size of 80 participants was chosen, taking into consideration lessons learned in prior studies. First, based on prior clinical studies at our site with biomechanical assessments, we anticipated a possible loss to follow-up of 15% [51, 52]. Second, we anticipated up to 15% of spinal stiffness measurements would be missing because participants were not able to tolerate the procedure [18]. Third, we expected 30% of FRP measurements would be missing due to insufficient signal-to-noise ratio in EMG measurements [53]. Given these factors, we anticipated obtaining a complete set of data from at least 50% of enrolled participants. We estimated 40 participants would be sufficient to obtain preliminary estimates of variability and effect sizes.

### Statistical analysis

Data analysis was done in the SAS System (Ver. 9.3, Cary, NC) for Windows. Descriptive statistics were calculated for all variables and Pearson correlation coefficients were also obtained between the physiological variables. First, to assess if the physiological variables changed over the course of treatment, mixed-effects regression models were fit for each of the physiological variables, over the 3 assessment time points (baseline, after 2 weeks, and after 6 weeks), adjusting for sex, age and body-mass index (BMI). Then, to assess if LBP outcome measures changed over the course of treatment, mixed-effects regression models were fit for each outcome, LBP intensity (VAS) and disability (RMDQ), over the 3 assessment time points. The effects of age, sex, and BMI were evaluated for each model and included if *p* ≤ 0.05. Separate models were then fit for the 2 outcomes and each of the physiological variables over the 3 assessment time points. The physiological variables were added to the models as random, time-varying covariates with all available data for the respective variable. The effect of which of the 2 EMG measurement systems was also evaluated in the FRP models and included if *p* ≤ 0.05. Model assumptions were examined with residual plots.

An exploratory analysis was done based on the recent findings using the same automated indenter device that we used [20]. Each participant who completed the study was categorized as a responder if they improved at least 30% from baseline on the RMDQ after 6 weeks or a nonresponder if they did not. We then fit 3 mixed-regression models, one for the automated lumbar spine stiffness, one for the hand-held stiffness at L3 and one for the palpatory stiffness at L3, over the 3 assessment time points with the binary responder variable adjusted for age, sex and BMI.

## Results

**Fig. 1:**
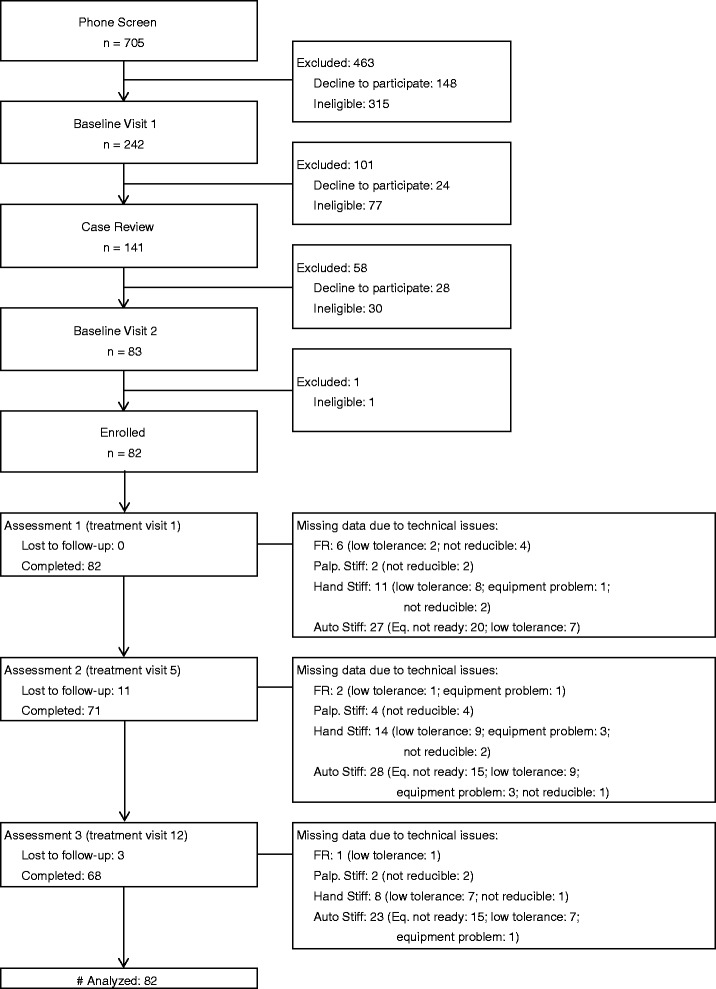
Study CONSORT flow diagram. FR: flexion relaxation; Palp. Stiff: had palpation stiffness assessment; Hand Stiff: hand-held device stiffness assessment; Auto Stiff: automated indenter stiffness assessment; Eq. not ready: equipment upgrade was needed for an unexpected safety concern, not ready for testing

Participant enrollment started in September 2012 and ended in May 2014, with data collection completed in June 2014. Figure 1 summarizes the study flow. Of 705 potential participants completing the phone screen, 242 were eligible for a BL1 on-site interview and physical examination, and 141 were further eligible for case review following the BL1 visit. A total of 82 participants were enrolled with 14 lost to follow-up by the end of study. All data available from the 82 participants were included in data analysis. The reasons for missing data due to technical issues are described in detail at each assessment visit in Fig. 1. The major reason for missing data for the spinal stiffness assessed using the automated indenter was that an equipment upgrade was necessary for an unexpected safety concern; the equipment was upgraded and used after the first 20 participants. Other main reasons for missing data included participants not able to tolerate test procedures due to increasing pain, equipment problems leading to data not being recorded, and data collected but not reducible.

Table 1 summarizes the demographic and health status of participants at baseline. The mean (SD) age was 44.9 (10.6) years and 39 participants (48%) were female. Most participants (84%) reported LBP for 1 year or longer in duration. The mean VAS was 46.1 mm (18.1) and the mean RMDQ score was 9.5 (4.3) at baseline. Table 2 summarizes the physiological variables and LBP intensity and disability at the baseline, week 2 and week 6 assessment time points.

**Table 1:** Descriptive statistics of selected demographic and health status variables (*n* = 82)

	*n*	%
Gender - female	39	48
Age (years), in mean (SD)	44.9	(10.6)
Body mass index, in mean (SD)	30.6	(5.3)
Race		
White	70	85
Black or African American	8	10
American Indian/Alaska Native	3	4
Asian	1	1
Ethnicity - Hispanic or Latino	5	6
Education		
High school graduate or less	17	21
Some college education or college degree	62	76
Post-graduate education or professional degree	3	4
Marital status -		
Married or living with partner	48	59
Divorced, separated, or widowed	17	21
Never married	16	20
Currently employed	60	73
Household Income – less than $40,000	39	48
QTF Diagnostic classification		
1: Pain without radiation	55	67
2: Pain + radiation to proximal extremity	19	23
3: Pain + radiation to distal extremity	8	10
Duration of current low back pain episode		
More than 12 weeks to 6 months	7	9
More than 6 months to 1 year	6	7
More than 1 year	69	84
Have seen a doctor of chiropractic	70	85
Beck Depression Inventory		
Minimal	69	84
Mild	8	10
Moderate	5	6
Physical activity		
None in the past month	25	30
Less than once a week	14	17
Once a week	7	9
2 to 3 times per week	21	26
4 or more times per week	15	18
Currently smoking cigarettes	25	30

*SD* standard deviation, *QTF* Quebec Task Force Classification for Spinal Disorders

**Table 2:** Descriptive statistics of physiological and patient-reported outcome variables at 3 assessment time points

		Baseline			After 2 Weeks			After 6 Weeks		
		n	Mean	SD	n	Mean	SD	n	Mean	SD
Physiological outcome variables										
Lumbar spine stiffness										
Palpatory	GS (N/mm) at L3	80	4.8	1.8	67	4.3	1.8	66	4.2	1.3
	GSV (N/mm)	80	2.3	1.5	67	2.0	1.1	66	1.9	1.1
	nGSV	80	0.5	0.2	67	0.5	0.2	66	0.5	0.2
Hand-held	GS (N/mm) at L3	69	7.7	2.1	57	7.5	1.8	59	7.6	2.2
	GSV (N/mm)	71	2.3	1.3	58	2.1	1.1	59	2.1	1.3
	nGSV	71	0.3	0.1	58	0.3	0.1	59	0.3	0.1
Automated	GS (N/mm) at Ant.	55	5.2	1.5	43	5.3	1.4	45	5.3	1.5
Flexion-relaxation ratio (FRR)										
Flexion FRR		76	4.7	3.7	69	5.8	5.4	66	5.5	5.6
Extension FRR		76	9.2	9.5	69	10.4	11.6	66	9.4	10.5
Asymmetry in flexion FRR		76	1.5	0.5	69	1.4	0.5	66	1.4	0.4
Asymmetry in extension FRR		76	1.4	0.7	68	1.4	0.4	65	1.4	0.5
Patient-reported outcome variables										
Average LBP during last 24 h	VAS (mm)	82	46.1	18.1	70	34.2	21.7	68	25.6	25.4
Roland Morris Disability Questionnaire		82	9.5	4.3	70	6.9	4.3	68	4.8	4.0

*SD* standard deviation, *GS* global stiffness, *GSV* global stiffness variation among five lumbar segments, *nGSV* normalized global stiffness variation (unitless), *Ant.* the most anterior point of the lumbar curvature in a prone posture, *FRR* flexion-relaxation ratio (unitless) pooled together for both EMG acquisition systems, *VAS* visual analog scale

Table 3 summarizes the correlations between spinal stiffness obtained using 3 assessment methods and the correlations between FRR and spinal stiffness within visits. The baseline GS obtained using the hand-held device at L3 was highly correlated with the GS obtained using the automated indenter at the most anterior point of the lumbar curvature at all assessment time points, but both were less correlated with GS obtained during hand palpation at L3. Correlations between the FRRs and the spinal stiffness measures were weak.

**Table 3:** Pearson’s correlations (*r*) between spine stiffness obtained using 3 methods and flexion-relation ratios

		Lumbar spine stiffness									
		Palpatory GS at L3		Palpatory GSV		Hand-held GS at L3		Hand-held GSV		Automated GS at Ant.	
	Visit	n	Pearson’s r	n	Pearson’s r	n	Pearson’s r	n	Pearson’s r	n	Pearson’s r
Lumbar spine stiffness											
Palpatory GS at L3	BL	80	-	80	0.45	67	0.46	69	0.29	53	0.42
	W2	67	-	67	0.59	53	0.47	54	0.19	41	0.30
	W6	66	-	66	0.32	57	0.47	57	0.03	45	0.15
Palpatory GSV	BL			80	-	67	0.11	69	0.16	53	0.08
	W2			67	-	53	0.23	54	0.28	41	0.17
	W6			66	-	57	0.18	57	0.28	45	0.04
Hand-held GS at L3	BL					69	-	69	0.39	51	0.86
	W2					57	-	57	0.21	39	0.63
	W6					59	-	59	0.38	44	0.81
Hand-held GSV	BL							71	-	52	0.37
	W2							58	-	40	0.05
	W6							59	-	44	0.31
Automated GS at Ant.	BL									55	-
	W2									43	-
	W6									45	-
Flexion-relaxation ratio											
Flexion FRR	BL	74	-0.16	74	0.05	63	0.03	65	−0.15	52	-0.10
	W2	65	0.07	65	0.04	56	0.12	57	−0.09	43	-0.21
	W6	64	-0.02	64	−0.22	57	0.20	57	−0.10	43	0.16
Extension FRR	BL	74	-0.14	74	−0.03	63	0.05	65	−0.14	52	-0.03
	W2	65	0.07	65	−0.01	56	0.16	57	0.03	43	-0.12
	W6	64	-0.01	64	−0.22	57	0.10	57	−0.11	43	−0.07
Asymmetry in flexion FRR	BL	74	0.05	74	0.04	63	0.07	65	0.30	52	-0.01
	W2	65	0.04	65	0.09	56	−0.11	57	0.19	43	0.08
	W6	64	0.05	64	0.11	57	0.15	57	0.15	43	0.02
Asymmetry in extension FRR	BL	74	0.08	74	0.00	63	0.08	65	0.11	52	0.09
	W2	64	0.05	64	0.02	55	0.12	56	0.32	43	0.07
	W6	63	0.10	63	0.05	57	0.20	57	0.21	43	0.11

*GS* global stiffness, *L3* the 3rd lumbar segment, *Ant.* the most anterior point of the lumbar curvature in a prone posture, *GSV* global stiffness variation among five lumbar segments, *nGSV* normalized global stiffness variation (unitless), *FRR* flexion-relaxation ratio (unitless) pooled together for both EMG acquisition systems, *BL* baseline, *W2* after 2 weeks, and *W6* after 6 weeks

The models for each of the physiological variables over the 3 time points, adjusted for sex, age and BMI, are given in Table 4. There was no change over time in the hand-held or automated lumbar spine stiffness measures, nor in the FRRs. There was a suggestion of small changes over time in the palpatory stiffness measures.

**Table 4:** Estimated regression coefficients from separate mixed-effects regression models of physiological covariates over time

Physiological variables^a^		Est.	SE	*p*
Lumbar spine stiffness				
Palpatory				
GS (N/mm) at L3	Intercept	5.51	0.71	<0.001
	Visit	−0.048	0.020	0.02
GSV (N/mm)	Intercept	0.84	0.62	0.18
	Visit	−0.036	0.018	0.04
nGSV	Intercept	0.13	0.11	0.27
	Visit	−0.003	0.003	0.29
Hand-held				
GS (N/mm) at L3	Intercept	13.28	0.83	<0.001
	Visit	−0.003	0.025	0.91
GSV (N/mm)	Intercept	1.78	0.65	0.01
	Visit	−0.014	0.019	0.47
nGSV	Intercept	0.05	0.08	0.52
	Visit	−0.002	0.002	0.47
Automated				
GS (N/mm) at Ant.	Intercept	8.49	0.79	<0.001
	Visit	0.006	0.022	0.78
Flexion-relaxation ratio				
Flexion FRR	Intercept	16.42	2.45	<0.001
	Visit	0.091	0.073	0.22
Extension FRR	Intercept	41.65	5.20	<0.001
	Visit	0.036	0.150	0.81
Asymmetry in flexion FRR	Intercept	1.38	0.29	<0.001
	Visit	−0.008	0.008	0.33
Asymmetry in extension FRR	Intercept	1.70	0.30	<0.001
	Visit	−0.001	0.009	0.89

*GS* global stiffness, *L3* the 3rd lumbar segment, *Ant.* the most anterior point of the lumbar curvature in a prone posture, *GSV* global stiffness variation among five lumbar segments, *nGSV* normalized global stiffness variation (unitless), *FRR* flexion-relaxation ratio (unitless) pooled together for both EMG acquisition systems, *Est.* estimated regression coefficients from corresponding regression models over 3 assessment time points (at baseline, after 2 weeks, and after 6 weeks), *SE* standard error

^a^All models adjusted for sex, age and BMI

Age and sex were not significantly related to VAS or RMDQ over the time assessments. BMI was not significantly related to VAS, but was significantly related to RMDQ. The average weekly improvement in VAS was 3 mm (parameter estimate per visit = −1.5 mm, SE = 0.2 mm, *p* < 0.001), with a mean reduction in LBP intensity (95% confidence interval) from baseline to week 6 of 20.1 mm (14.1 to 26.1). The average weekly improvement in RMDQ was 0.8 points (parameter estimate per visit = −0.4, SE = 0.04, *p* < 0.001), with a mean reduction in LBP-related disability adjusted for BMI of 4.8 (3.7 to 5.8) at week 6.

As can be seen in the separate regression models given in Table 5, the addition of each physiologic variable did not affect the parameter estimates for LBP intensity or LBP-related disability over time. There was no relationship seen between physiological variables and LBP intensity. However, higher levels of hand-held GS at L3 and automated GS were statistically significantly associated with higher levels of RMDQ and lower levels of flexion and extension FRR were significantly associated with higher levels of RMDQ across the 3 assessment time points.

**Table 5:** Estimated regression coefficients from separate mixed-effects regression models of pain intensity and disability

		Pain intensity(visual analog scale, mm)			Disability score^a^(Roland Morris Disability Questionnaire)		
Physiological variables		Est.	SE	*p*	Est.	SE	*p*
Lumbar spine stiffness							
Palpatory	Intercept	41.9	4.5	<0.001	2.0	2.7	0.45
	Visit	−1.4	0.2	<0.001	−0.4	0.04	<0.001
	GS (N/mm) at L3	0.7	0.9	0.44	0.3	0.2	0.09
	Intercept	42.9	3.0	<0.001	3.5	2.4	0.15
	Visit	−1.4	0.2	<0.001	−0.4	0.04	<0.001
	GSV (N/mm)	1.0	1.0	0.35	0.3	0.2	0.15
	Intercept	43.6	3.4	<0.001	3.8	2.4	0.11
	Visit	−1.4	0.2	<0.001	−0.4	0.04	<0.001
	nGSV	3.0	5.6	0.60	1.2	1.1	0.27
Hand-held	Intercept	45.4	6.6	<0.001	0.1	3.7	0.98
	Visit	−1.8	0.2	<0.001	−0.4	0.05	<0.001
	GS (N/mm) at L3	0.03	0.8	0.97	0.5	0.2	0.02
	Intercept	47.7	3.3	<0.001	7.6	2.3	0.002
	Visit	−1.8	0.2	<0.001	−0.4	0.05	<0.001
	GSV (N/mm)	−1.0	1.2	0.39	−0.1	0.2	0.59
	Intercept	47.1	3.5	<0.001	7.9	2.2	<0.001
	Visit	−1.8	0.2	<0.001	−0.4	0.05	<0.001
	nGSV	−5.8	10.1	0.57	−2.7	2.1	0.20
Automated	Intercept	52.3	6.8	<0.001	−2.1	4.3	0.62
	Visit	−1.4	0.3	<0.001	−0.4	0.05	<0.001
	GS (N/mm) at Ant.	−1.5	1.2	0.23	0.7	0.3	0.03
Flexion-relaxation ratio							
Flexion FRR	Intercept	44.7	2.6	<0.001	10.0	0.6	<0.001
	Visit	−1.6	0.2	<0.001	−0.4	0.04	<0.001
	FRR	0.08	0.3	0.81	−0.2	0.06	0.02
Extension FRR	Intercept	45.2	2.6	<0.001	10.0	0.6	<0.001
	Visit	−1.5	0.2	<0.001	−0.4	0.04	<0.001
	FRR	0	0.2	0.96	−0.1	0.03	0.008
Asymmetry in flexion FRR	Intercept	44.1	3.7	<0.001	9.0	0.8	<0.001
	Visit	−1.5	0.2	<0.001	−0.4	0.04	<0.001
	FRR	0.7	2.2	0.76	0.1	0.43	0.75
Asymmetry in extension FRR	Intercept	42.6	3.8	<0.001	8.3	0.8	<0.001
	Visit	−1.6	0.2	<0.001	−0.4	0.04	<0.001
	FRR	1.9	2.3	0.42	0.6	0.5	0.16

*GS* global stiffness, *L3* the 3rd lumbar segment, *Ant.* the most anterior point of the lumbar curvature in a prone posture, *GSV* global stiffness variation among five lumbar segments, *nGSV* normalized global stiffness variation (unitless), *FRR* flexion-relaxation ratio (unitless) pooled together for both EMG acquisition systems, *Est.* estimated regression coefficients from corresponding regression models over 3 assessment time points (baseline, after 2 weeks, and after 6 weeks) on time-varying physiological covariates, *SE* standard error

^a^All palpatory stiffness models, hand-held GS and automated GS also adjusted for BMI

Of the 68 participants followed through week 6, 49 (72%) were classified as responders. The exploratory analysis showed that neither the spinal stiffness based on the automated (F_1,47_ = 0.08, *p* = 0.78) or hand-held device (F_1,60_ = 0.63, *p* = 0.43) was significantly related to responder status. Spinal stiffness based on the palpatory method was significantly related to responder status (F_1,63_ = 5.38, *p* = 0.02). The adjusted mean (SE) baseline stiffness of responders [4.6 (0.2)] was less than that of nonresponders [5.3 (0.3)], but the changes over time were not different.

There were no serious adverse events (AEs) reported in the study. A total of 63 AEs were categorized as “Possible/Probably/Definite” related to the study activities, including 6 during examination, 36 during treatment, 10 during biomechanical testing, and 11 attributed to both treatment and biomechanical testing. All these AEs were mild. Of those AEs unrelated to the study, 10 were moderate, and 3 were severe with two cases of fractured rib due to unrelated exercise activities and 1 case of tongue swelling leading to an ER visit.

## Discussion

In this study, we investigated the association between patient-reported pain intensity and disability and two sets of physiological variables (lumbar spine stiffness and FRP) in adults with chronic LBP who received twice-weekly, 6 weeks of thrust SM treatment. The results demonstrated clinically and statistically significant decrease in both pain intensity and disability following the treatment. We also observed that participants who showed a stiffer lumbar spine (e.g., higher values of GS at L3 obtained using the hand-held device) had a higher level of disability. Similarly, participants who showed less distinct lumbar FRP (e.g., lower levels of flexion FRR) had a higher level of disability. On the other hand, lumbar spine stiffness and FRP demonstrated no association with pain intensity. Regarding the effects of thrust SM on physiological variables, only spinal stiffness obtained during hand palpation decreased slightly over the course of treatment, while spinal stiffness obtained using the other two methods and FRR did not.

### Patient-reported outcomes

Our findings in patient-reported outcomes are consistent with previous reports that SM is an effective treatment option for patients with LBP [7, 8, 51, 54–56]. Particularly, the model estimated mean reduction in pain intensity was 20.5 mm in VAS, exceeding the literature recommended minimal clinically important difference (MCID) of 15 mm proposed by Ostelo et al. (2008) or 20 mm proposed by Hagg et al. (2003) [57, 58]. The model estimated mean reduction in RMDQ was 4.8, exceeding the literature recommended MCID of 2 to 3 points proposed by Bombardier et al. (2001) or at the borderline of the recommended MCID of 5 points proposed by Stratford et al. (1998) and Ostelo et al. (2008) [58–60]. The reduction in pain intensity and disability relative to baseline well exceeded the literature recommended MCID of 30% reduction from baseline proposed by Stratford et al. (1998) and Ostelo et al. (2008) [58, 60]. The positive pain and disability outcomes to SM treatment in the current study might be in part attributed to the inclusion/exclusion criteria based on the experience learned from previous studies conducted at our facility [56]. Specifically, only participants who had a disability score of at least 6 in RMDQ were recruited. Additionally we employed a 6-week, 12-visit treatment scheme, consistent with the most recent finding of optimal SM treatment length for chronic LBP management [61].

### Lumbar spine stiffness

Our findings in the association of spinal stiffness with patient-reported outcomes corroborate with one previous study on patients with chronic LBP who underwent SM treatment [22]. Ferreira et al. (2009) randomly allocated patients with chronic LBP to 3 treatment groups: SM (*n* = 71), motor control exercise (*n* = 60), and a general exercise program (*n* = 60). Spinal stiffness was assessed at the most symptomatic lumbar level using a modified manual assessment approach, in which the raters matched their subjective judgment of spinal stiffness against a stiffness reference device and rated accordingly using an 11 point scale. The authors found that baseline spinal stiffness was not associated with response to any of three types of treatment. Particularly, the regression coefficients (95% confidence interval) for the association between baseline spinal stiffness and pain intensity (VAS) and disability (RMDQ) were −0.40 (−0.95 to 0.15) and −0.04 (−1.13 to 1.05), respectively, for the SM group. Additionally the authors found no association between change in spinal stiffness and change in disability score.

There are several differences between the current study and the study by Ferreira et al. (2009). The selection of participants in the study by Ferreira et al. (2009) was less restrictive than those in the current study. We limited the study population to those with QTF classification of 1, 2, or 3, baseline RMDQ ≥6, and persistent baseline pain intensity ≥2. Additionally we used instrument-assisted, objective spinal stiffness assessment methods and reported results in a continuous scale, while Ferreira et al. (2009) used a semi-objective, 11-point, stiffness reference device-based assessment method. In the current study we only used side-lying thrust SM as treatment, while thrust SM or spinal mobilization was used at the discretion of the treating clinicians in the study by Ferreira et al. (2009). On the other hand, both studies employed 12 sessions of SM treatment that is consistent with the best current evidence for the optimal length of SM treatment [61]. Further spinal stiffness was assessed at the most symptomatic levels in the Ferreira et al. study (2009), while we examined GS preset at L3 in the current study. Given that a symptomatic segment can be hypermobile (and/or clinically unstable), hypomobile or have relatively normal mobility, the study by Ferreira et al. could face the same limitation as the current study. Nevertheless, the current study confirmed the observation made by Ferreira et al. (2009) that spinal stiffness doesn’t appear to be associated with patient-reported outcomes following SM treatment.

It is noteworthy that different results on spinal stiffness have been made in patients with acute LBP [20, 24]. Wong et al. (2015) recruited patients with acute LBP and categorized them by response to SM treatment (i.e., decrease in Oswestry Disability Index score ≥ 30% from baseline) [20]. Spinal stiffness was assessed using the automated indenter device of the same design as used in the current study. The authors found that responders had immediate decrease in spinal stiffness following treatment that was sustained at day 7. However, no change in spinal stiffness was observed in the nonresponders. In the current study, we were interested in the sustained changes in spinal stiffness over 6 weeks of treatment as our study population involved chronic LBP. Using a similar analysis described by Wong et al. (2015), we did not observe any sustained change in spinal stiffness assessed using the hand-held device or the automated device in the responders (i.e., decrease in RMDQ ≥30% from baseline). For spinal stiffness assessed during hand palpation, baseline stiffness of responders was less than that of nonresponders. However, the changes over time were not different between the two groups. Therefore, regardless of the assessment method, changes in spinal stiffness were not related to improvement in RMDQ. There are a number of differences between the study by Wong et al. (2015) and the current one. Besides the difference in LBP chronicity, patients recruited in Wong et al. (2015) were younger and leaner. Additionally the assessment interval was 7 days in Wong et al. (2015) compared to 2 weeks and 6 weeks in the current study. The assessment intervals were justified for both studies with respect to the patient population involved and the particular research questions asked. The lack of sustained change in spinal stiffness observed in the current study suggest that spinal stiffness in patients with chronic LBP behaves differently from those with acute LBP.

#### Instrument-assisted spinal stiffness assessment

In the current study we chose 3 instrument-assisted methods to assess spinal stiffness. We found larger mean GS values obtained using the hand-held device at L3 than GS obtained during hand palpation at L3 and GS obtained using the automated indenter at the spinous process closest to the most anterior point of the lumbar curvature (mainly at L3). On the other hand, GS obtained using the hand-held device was highly correlated with GS obtained using the automated device, while both were less correlated to GS obtained using palpation. Additionally we observed a statistically significant decrease, though small, in spinal stiffness at L3 obtained during hand palpation over the course of treatment, but not when using the hand-held device or the automated device. In contrast, spinal stiffness obtained using the latter two methods demonstrated that participants who had higher values of spinal stiffness were also those who had a higher level of disability. Such association was not observed when using spinal stiffness obtained during hand palpation. These inconsistent observations could be in part attributed to considerable differences between the three assessment methods. The area assessed is much larger during hand palpation (i.e., the base of the palm), thus exerting force on both the bony spine and the back muscles. The other methods strictly apply force on the spinous process. Additionally the rate of force application during the hand palpation and the hand-held device methods (roughly 1 repetition per second) was faster than the rate used in the automated indenter procedure (4–8 s dependent on participant subcutaneous adipose tissue thickness). Further, different breathing instructions were given to participants during 3 assessment methods. Finally, participants may respond differently to stiffness assessment when touched by the human hand versus mechanical devices.

While previous studies focused on examining spinal stiffness at individual levels, we also evaluated variations in lumbar spine stiffness (i.e., GSV and nGSV) as suggested by our study clinicians because they were perceiving the fluctuation in spinal stiffness across the entire lumbar spine and factored it in their report of perceived spinal stiffness. The correlation between GSV and GS was weak, suggesting the potential value of GSV as a separate variable from GS. However we did not observe any association of GSV or nGSV with patient-reported outcomes. More studies are needed to understand variations in lumbar spine stiffness and its clinical relevance.

### Flexion-relaxation phenomenon

In the current study, there was no change in FRR values observed over the course of SM treatment. In combination with previous study findings, there is limited value of FRP assessment in patients with chronic LBP, despite the observation that participants who showed less distinct lumbar FRP had a higher level of disability.

#### Association between flexion-relaxation phenomenon and spinal stiffness

It is well established that spinal muscles provide the spine its stiffness. It is possible that elevated spinal stiffness is a result of aberrant trunk muscle activity, in addition to potential structural and/or tissue property-related changes in the spine [31, 32]. However, the results from this study suggest that FRP and spinal stiffness were at most weakly associated (with correlation coefficients between 0.2 and 0.25). It possible that the activity of the paraspinal muscles was low during the spinal stiffness measurement settings in a prone posture (as opposing to active postural control), thus not affecting spinal stiffness substantially. Therefore, future studies should focus more on dynamic spinal stiffness during movement to understand the potential contribution of the altered neuromuscular control in patients with chronic LBP.

### Analgesic effect of spinal manipulation and the measured physiological variables

A number of acting mechanisms, involving biomechanical, neurophysiological, immunological, and/or neurohormonal pathways, have been proposed to explain the beneficial effects of SM [62–65]. Currently the most intensively studied is the analgesic effect of SM that is well illustrated by a comprehensive model proposed by Bialosky et al. (2009) [66]. Essentially, SM is considered as a form of mechanical stimulus to the spinal tissues that may trigger a cascade of neurophysiological responses from the body (e.g., ascending and descending pain inhibitory pathways) and eventually lead to pain relief. The physiological variables assessed in the current study are part of the trunk neuromuscular behavior that is susceptible to pain-related disability. For instance, patients with higher RMDQ showed a higher level of GS at L3 and a lower level of FRR. On the other hand, our study findings suggest at best small effects on the two physiological variables following 6 weeks of SM, despite significant improvement in LBP. Observations on other measures of trunk neuromuscular behavior in patients with LBP, such as postural sway, have also been reported in the literature. Ruhe et al. (2011) demonstrated a strong correlation between sway speed and pain intensity in participants with LBP [67]. They also observed that participants with pain improvement of at least 4 in NRS after 3 sessions of manual intervention (e.g., SM) showed significant decrease in sway speed, while those with improvement less than 4 did not [68]. On the other hand, patients with mostly chronic LBP did not show change in postural away after 2 weeks and 4 sessions of either thrust SM or nonthrust SM in a study by Goertz et al. (2016) [69]. Overall, while the relationship between nociception, neuromuscular behavior, and SM are very complex, the findings from the current study suggest lumbar spine stiffness and FRP are not substantially changed in association with LBP improvement following a course of SM.

### Limitations

This study was designed as a single-arm trial to measure how lumbar spine stiffness and FRP related to clinical outcomes in adults with chronic LBP. Though more rigorous study design such as randomized controlled trial is necessary to examine the role of these two physiological variables in the observed clinical improvement following SM, the findings of the current study appear to refute the possibility of such role in the case of chronic LBP. Some limitations need to be considered for the current study. GS is obtained by measuring deformation across the entire lumbar spine resembling a cantilever beam. In other words, GS is not a measure of intersegmental stiffness. Its clinical validity is currently under investigation [20, 24]. Another limitation is the switch in the surface EMG acquisition system at the early stage of the trial for FRP assessment. However, no significant effect was observed for between the 2 equipment set-ups. Furthermore, caution needs to be taken for the generalization of the current study findings. First, the study population was screened based primarily on the participant’s disability level. This may in part be attributed to the positive observations associated with disability scores but not with pain intensity. Second, the application of the findings should be restricted to chronic LBP populations. A number of previous studies have demonstrated clinical values of spinal stiffness assessment for patients with acute LBP [24, 70–72]. Such discrepancy could be attributed to the differences in underlying pathophysiologic mechanisms between acute and chronic LBP, in which tissue injury plays a key role in acute LBP while central sensitization plays a key role in chronic LBP, in addition to psychosocial factors contributing to the two conditions [73–76]. Third, other factors such as inclusion/exclusion criteria, duration of treatment, and timing of assessment should be considered when comparing results across different studies. Fourth, modified side-lying thrust SM was used as the intervention to facilitate segmental load estimation during TV1, TV5, and TV12. This may affect effectiveness of SM treatment and outcomes. Finally, three DCs rendered treatment decisions for participants with chronic LBP attributed to different causes and which presented with varied symptom characteristics. Clinical decision-making was based on many individual factors including the primary diagnosis, positions of relief and provocation, participant tolerance, clinical history, personal experience and preferences. These complex clinical decisions resulted in some differences in SM delivery. The variation in treatment delivered, as well as the limited treatment duration (6 weeks) and frequency (12 sessions), may contribute to the small changes in spinal stiffness despite significant improvements in pain intensity and disability.

## Conclusions

In the current study we examined the lumbar spine stiffness and FRP in participants with chronic LBP who underwent 12 sessions of thrust SM over 6 weeks. We observed both clinically and statistically significant improvement in both pain intensity and disability, but only small changes in GS and FRR following the treatment. Despite that participants who showed a stiffer lumbar spine, as well as those who showed a less distinct FRP, had a higher level of disability, improvement in pain and disability observed in study participants with chronic LBP was not associated with the measured GS or FRR. These findings may be in part attributed to varied clinical conditions causing LBP, subsequent clinical decision making, and therefore SM treatment delivered over a limited visit duration and frequency. Studies further standardizing clinical decision making among clinicians and including longer treatment duration and follow-up periods should be considered.

## Abbreviations

AE: Adverse event
BL1: Baseline Visit 1
BL2: Baseline Visit 2
EMG: Electromyography
FRP: Flexion-relaxation phenomenon
FRR: Flexion-relaxation ratio
GSV: Global stiffness variation
IRB: Institution Review Board
L3: the third segment of the lumbar spines
LBP: Low back pain
MCID: Minimal clinically important difference
nGSV: Normalized global stiffness variation
NIH: National Institute of Health
PA: Posterior-anterior
QTF: Quebec Task Force (Classification for Spinal Disorders)
RMDQ: Roland Morris Disability Questionnaire
SD: Standard deviation
SE: Standard error
SM: Spinal manipulation
TV: Treatment visit
VAS: Visual analog scale
